# Effects of Different Tillage Measures on Soil Physical Properties, Organic Carbon Sequestration and Crop Production in Reclaimed Farmland Filled with Foreign Soil

**DOI:** 10.3390/plants15081239

**Published:** 2026-04-17

**Authors:** Xinsheng Wang, Jiaju Dong, Shouchen Ma, Zhenhao Gao, Huihao Liu, Shoutian Ma

**Affiliations:** 1 School of Surveying and Land Information Engineering, Henan Polytechnic University, Jiaozuo 454000, China; hpuwxs@126.com (X.W.); jiajudong@yeah.net (J.D.); hygge010309@163.com (Z.G.); lhh9528@163.com (H.L.); 2 School of Agriculture, Henan Institute of Science and Technology, Xinxiang 453003, China; 3 Institute of Farmland Irrigation, Chinese Academy of Agricultural Sciences (CAAS)/Key Laboratory for Crop Water Requirement and Regulation, Ministry of Agriculture and Rural Affairs of China, Xinxiang 453002, China; 4 Field Observation and Research Station of Efficient Water Use for Agriculture, Xinxiang 453002, China

**Keywords:** tillage methods, land reclamation, filled with foreign soil, soil porosity, soil carbon sequestration

## Abstract

A long-term positioning experiment was conducted from 2014 to 2021 to determine the appropriate tillage method for rapidly improving soil quality in reclaimed land. Four tillage methods were arranged before winter wheat sowing: deep tillage (DT), shallow tillage (ST), DT-ST alternate rotation (DST) and no tillage (NT). The results showed that: (1) with increasing reclamation years, ST, DT and DST had lower soil bulk density (SBD) and higher soil total porosity (STP) and soil capillary porosity (SCP) compared to NT. In the early stage of reclamation, ST had the lowest SBD and the highest STP and soil non-capillary porosity (NCP) in 0–20 cm soil layer, DT had the highest SCP and lowest NCP. In the 20–40 cm soil layer, DT has the lowest SBD and highest STP and SCP, followed by DST. In the late stage, SBD of each soil layer was NT > ST > DT > DST, while STP and SCP were NT < ST < DT < DST. (2) Different tillage methods influenced soil organic carbon (SOC) accumulation by affecting carbon sequestration rate (CSR). As opposed to NT, DT rapidly increased SOC of 0–40 cm soil layer in the early stages of reclamation, whereas DST facilitates maintaining higher SOC in the later stages. As compared to DT and DST, ST contributed more to SOC accumulation in surface soil, but less to SOC accumulation in deep soil. (3) Different tillage methods had various influences on SOC stratification ratio (SR). During the initial reclamation stage, NT had the lowest SR. Nevertheless, NT and ST maintained their high SR in the subsequent stage, whereas the SR of DT and DST experienced a notable decline due to the increase in SOC in deep soil. (4) It was observed that ST, DT and DST had higher grain yields compared with NT. The correlation analysis showed that DT improved soil properties by promoting SOC accumulation, increasing SCP and reducing NCP, thus increasing grain yield in the early stage of reclamation, while in the later stage of reclamation, DST can maintain better soil quality by reducing SBD and maintaining higher STP, SCP and SOC, and balanced the reasonable distribution of soil nutrients between the upper and lower soil layers by reducing SR of SOC, which helps the crop to maintain higher grain yields over time.

## 1. Introduction

It is true that coal mining contributes greatly to the national economy, but it also negatively impacts the region’s ecology, production, and living environment. In particular, mining subsidence has caused serious damage to cultivated land, directly affecting regional crop production and resulting in a large amount of cultivated land being deserted. Therefore, it is of great practical value to reclaim the damaged land in mining areas to improve regional ecological condition and ensure sustainable food production. As for the reclamation of damaged land in mining areas, relevant studies mainly focus on reclamation technology, vegetation restoration mode and the quality of reclaimed land and so on [1,2], and seldom pay attention to the influence of field management measures on the quality of cultivated land after reclamation, and especially on rational tillage measures of reclaimed land. Suitable tillage methods can improve soil quality and contribute to sustainable crop production. Long-term irrational tillage (such as deep tillage) will damage soil structure and accelerate soil organic carbon (SOC) loss, resulting in the thinning of topsoil and decreased soil fertility [3,4]. However, long-term no tillage and shallow tillage will lead to the enrichment and stratification of organic carbon and nutrients on the surface [5,6], and also easily cause soil compaction and poor permeability, which will affect the growth and development of crop roots [7,8]. Therefore, exploring the effects of different tillage methods on the soil structure and soil quality of reclaimed land can provide an important theoretical basis for the selection of scientific and reasonable field management methods of reclaimed land, so as to rapidly improve the quality of reclaimed soil.

Soil bulk density (SBD) and soil porosity have an important effect on soil moisture, fertilizer, air, temperature, nutrient transport and microbiological characteristics, which are closely related to seed germination, seedling quality and root growth [9]. The influence of tillage on soil structure is firstly reflected in the changes in SBD and soil porosity. Different tillage methods have various degrees of soil disturbance, resulting in great differences in the direction and degree of impact on SBD. Tillage also directly affects the total porosity (STP), capillary porosity (SCP) and non-capillary porosity (NCP) of soil. The quantity and proportion of different pores in soil affect the soil water, air, temperature and other factors. In order to meet the needs of crops for soil moisture and air, the soil, especially soil in plowing layer, should not only have proper amount of pores, but should also have a suitable proportion of different sizes of pores. SOC plays an important role in soil structure and soil biodiversity, thereby affecting soil water and fertilizer retention performance, soil fertility and crop yield. Therefore, SOC is often used to evaluate soil quality and health [10]. Tillage measures have important effects on soil physical structure by influencing SOC content. Conversely, soil structural characteristics such as SBD and soil porosity also affect SOC sequestration in the soil. Soil with higher SBD has lower soil porosity and poor soil permeability and ventilation, which are not conducive to root growth and soil microbial survival, thus affecting SOC accumulation [11]. Therefore, in the process of land reclamation, this practical problem needs to be solved urgently in order to choose the correct tillage method to improve soil structure and increase soil carbon sequestration capacity and soil fertility.

At present, the area of land destroyed by coal mining in China reaches 1.5 × 10^7^ ha, which is still increasing at a rate of 4 × 10^5^ ha per year. In order to improve the agricultural production environment and maintain regional food security, the local government initiated land reclamation in the mining area that is the focus of this research, where large-scale coal mining had caused severe land subsidence, cultivated land damage, and farmland abandonment. In the process of land reclamation, due to the large amount of soil disturbance caused by engineering measures, the soil structure of reclamation land is poor, and soil nutrients, especially SOC, are seriously insufficient, which limits the rapid recovery of productivity of reclaimed land. A reasonable tillage method combined with straw returning can not only improve SOC level, thereby improving soil physical structure, but can also increase soil nutrient contents, providing abundant nutrients for crop growth [12,13]. Therefore, in this study, a long-term positioning experiment of 8 years was conducted to discover a suitable tillage method that contributes to the rapid improvement of the reclaimed soil quality by comparing the soil characteristics and yield performance of reclaimed land under different tillage measures, consequently providing a theoretical basis and technical support for land reclamation in mining areas. Based on the above considerations, we hypothesized that (i) different tillage methods would significantly affect soil structure and SOC dynamics in reclaimed farmland; (ii) different tillage methods would show contrasting effects on SOC accumulation at different reclamation stages; (iii) changes in SOC would be closely related to soil physical properties such as bulk density and porosity.

## 2. Materials and Methods

### 2.1. Overview of the Study Area and Experimental Design

The research area is located in Zhaogu coal mine, Huixian City, Henan Province, China (35°23′–35°28′ N, 113°33′–113°57′ E). It is an important wheat and maize double cropping production area in China. Large-scale coal mining has seriously damaged the local land resources, and a large number of cultivated lands have been abandoned due to mining subsidence. In order to improve the ecological condition of the mining area and alleviate the contradiction between people and land, the local government began to reclaim land damaged by mining subsidence in 2012. The reclaimed land that serves as the object of research was originally an abandoned village. In 2012, it was reclaimed and turned into arable land by filling the areas with foreign soils, at a thickness of 50–60 cm. The filling soils were sourced from nearby grassy fields, and soil samples (0–60 cm) were collected before wheat sowing using a five-point sampling method to determine the basic physical and chemical properties of the reclaimed soil (Table 1). The experiment was conducted from 2013 to 2021. The experimental period (2014–2021) was divided into an early stage (2014–2017) and a late stage (2018–2021). Before winter wheat sowing, 750 kg ha^−1^ of N, P and K compound fertilizer (N:P_2_O_5_:K_2_O = 15:15:15) was applied, and four tillage methods, including deep tillage (DT), shallow tillage (ST), DT–ST alternating tillage (DST), and no tillage (NT), were set up on the basis of straw returning. Each treatment had three replicates, resulting in 12 plots, each with an area of 300 m^2^. N topdressing was applied at 120 kg ha^−1^ at the jointing stage. At the wheat maturity stage, plant samples were first collected for grain yield measurement. After that, soil samples of 0–40 cm soil layer were collected at 20 cm intervals and brought back to the laboratory for soil physical and chemical properties measurement. Three duplicate soil samples were collected for each treatment.

### 2.2. Monitoring Items and Methods

SBD and soil porosity. After winter wheat harvest, soil samples of 0–20 cm and 20–40 cm soil layers were collected in each plot with a ring cutter, and brought back to the laboratory to measure SBD, soil total porosity (STP), capillary porosity (SCP) and non-capillary porosity (NCP).

Soil organic carbon (SOC). At the wheat maturity stage, the soil samples of the 0–40 cm soil layer were collected at 20 cm intervals with a soil drill and brought back to the laboratory for SOC measurement. SOC was determined by potassium dichromate volume–external heating method [14].

Soil carbon sequestration rate (CSR). CSR is calculated according to the following formula:

(1) CSR (t C·ha^−1^·a^−1^) = (CCS − ICS)/T

where CCS is current SOC storage; ICS is initial SOC storage; T(a) is time interval between two tests. SOC storage (t C ha^−1^) in each soil layer was calculated using SOC concentration, soil bulk density, and soil layer thickness.

Stratification ratio (SR) of SOC. SR is the ratio of SOC in the top layer to that in lower layer, which reflects changes in soil quality resulting from various tillage methods. As a result, it can be used to evaluate soil succession directions [4]. In this study, the ratio of SOC in 0–20 cm soil layer to that in 20–40 cm soil layer was used as the SR of SOC.

Determination of grain yield. At the wheat maturity stage (BBCH 89), plant samples of 1.0 m^2^ were collected for grain yield measurement in test years.

### 2.3. Statistical Analysis Methods

SPSS 25.0 software was used for statistical analysis, and an LSD test was used to compare the differences among different tillage methods (*p* < 0.05).

## 3. Results and Analysis

### 3.1. SBD and Soil Porosity Under Different Tillage Methods

**Figure 1:**
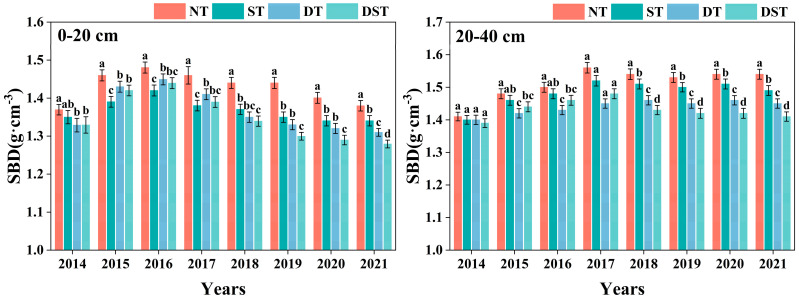
Changes in SBD in different tillage methods. NT: no tillage; DT: deep tillage; ST: shallow tillage, DST: DT-ST alternating tillage; SBD: soil bulk density. Different small letters show significant differences among treatments (*p* < 0.05).

**Figure 2:**
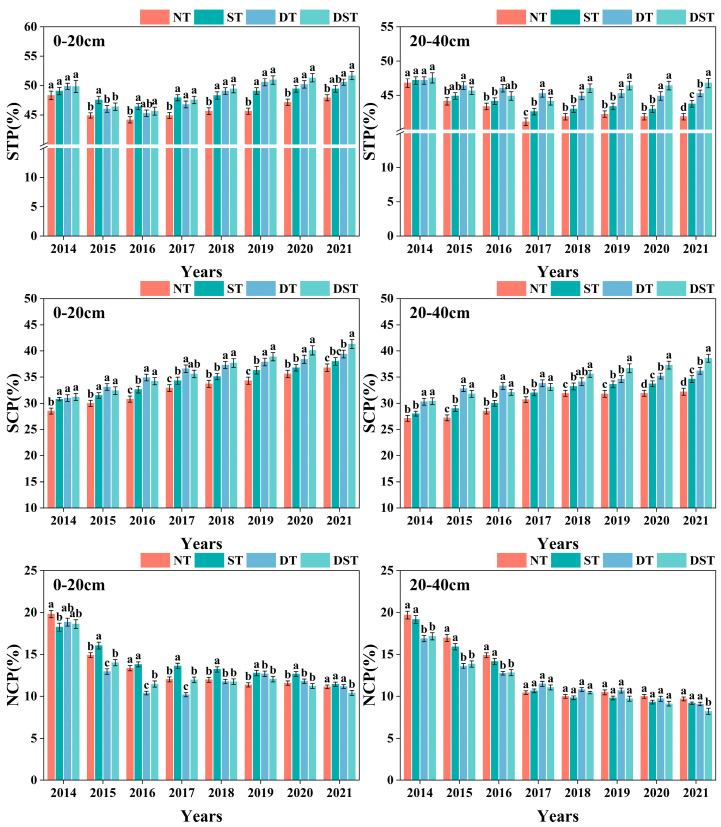
Changes in STP, SCP and NCP in different tillage methods. NT: no tillage; DT: deep tillage; ST: shallow tillage, DST: DT-ST alternating tillage; STP: Soil total porosity; SCP: soil capillary porosity; NCP: soil non-capillary porosity. Different letters in the same year show significant differences among treatments (*p* < 0.05).

As part of the land reclamation process, engineering measures such as excavation, transportation, filling and flattening have a great influence on the structure of the filled soil. As a result, the soil aggregate structure of reclaimed farmland can be damaged and the soil can come loose, resulting in a temporary decrease in SBD and increase in STP and NCP. With the increase in reclamation years, the SBD of all reclaimed farmland first increased and then decreased, the STP first decreased and then increased, and the SCP gradually increased, while the NCP rapidly decreased and tended to be stable. Different tillage measures had various effects on the SBD of each soil layer in different reclamation periods. In the early stage of reclamation (2014–2017), ST had the lowest SBD in 0–20 cm soil layer, followed by DST. In the 20–40 cm soil layer, DT had the lowest SBD, followed by DST (Figure 1). But in the late period (2018–2021), the SBD of each soil layer was NT > ST > DT > DST. DST had the lowest SBD in all soil layers (Figure 1). With the increase in reclamation years, the STP and SCP of each treatment were significantly higher than those of NT. The influence of different tillage methods on the soil porosity of each soil layer also varied in different periods. In the early stage of reclamation, ST had the highest STP and NCP in the 0–20 cm soil layer, and DT had the highest SCP and the lowest NCP. DT has the highest STP and NCP in the 20–40 cm soil layer, followed by DST. In the late reclamation period, the STP and NCP of each soil layer were NT < ST < DT < DST. DST had the highest STP and NCP in all soil layers (Figure 2). It can be seen that DT rapidly improves soil structure by increasing SCP and reducing NCP of each soil layer in the early stage. With the increase in reclamation years, DST helps to maintain lower SBD and higher STP and SCP in each soil layer, so that it had better soil structure characteristics in the late stage of reclamation.

### 3.2. Changes in SOC Contents in the Reclaimed Soil

In the early stage of reclamation (2014–2017), the SOC content of each treatment increased rapidly with the increase in reclamation years, and tended to be stable in the late stage (2018–2021). Tillage methods had different effects on SOC in different soil layers. In the early stage of reclamation, the SOC content of each soil layer under DT and DST was greater than that of NT and ST, and DT had the highest SOC content. In the late stage, ST had the highest SOC content in 0–20 soil layer, followed by DST. The SOC content of DST and DT was significantly higher than that of ST and NT in 20–40 cm soil layers, and DST had the highest SOC content (Table 2). It is concluded that DT accelerates the rapid rise in SOC within each soil layer at the beginning of reclamation. ST increased the SOC content in the surface soil, but had little effect on the SOC in the deep soil compared with DT, and long-term DST contributed to the increase in the SOC content of the whole soil profile. The variation in SOC storage in the 0–20 cm layer was generally consistent with that of SOC concentration (Table 3). In the early stage of reclamation (2014–2017), DT had the highest SOC storage in the 0–20 cm layer, followed by DST, while NT had the lowest SOC storage. In the later stage (2018–2021), ST maintained the highest SOC storage in the surface soil, indicating its stronger capacity for topsoil carbon retention under long-term management.

### 3.3. Effects of Tillage Methods on CSR and SR

Different tillage measures had various effects on CSR of different soil layers. In the early stage of reclamation (2014–2017), DT had the highest CSR, followed by DST, and NT had the lowest CSR. At the later stage (2018–2021), NT had the highest CSR in 0–20 cm soil layer, followed by ST, and DT had the lowest CSR. In the soil layer of 20–40 cm, DST had the highest CSR, followed by ST (Table 4). Different tillage measures had different effects on SR of SOC. During 2014–2017, the SR of all treatments showed an increasing trend, and NT had the lowest SR (Table 5). With the increase in reclamation years, NT and ST maintained a high SR, DT and DST significantly increased SOC in deep soil, and so reduced their SR, and DT had the lowest SR of SOC in the late reclamation period (2018–2021).

### 3.4. Effects of Different Tillage Measures on Productivity of Reclaimed Land

The grain yield of each treatment increased rapidly with the increase in reclamation years in the early stage of reclamation (2014–2017). After this (2018–2021), the grain yield of ST, DT and DST tended to be stable with varying degrees of fluctuation, but that of NT was still in a slow increase trend. Different tillage methods have various effects on yield at different stages (Table 6). In the early stage of the reclamation, ST, DT and DST had higher yield than NT, and DT had the highest yield. In the later stage of reclamation, DST had the highest yield, while DT had lower yield compared with DST. The correlation analysis demonstrated that crop yield was significantly positively correlated with SCP_20_, SOC_20_, SOC_40_, SOC_T_, SR and negatively correlated with STP_20_, STP_40_, NCP_20_, and NCP_40_ in the early stage of reclamation. By the late growth period, the yield was significantly positively correlated with STP_20_, STP_40_, SCP_20_, SCP_40_, SOC_20_, SOC_40_, and SOC_T_, and significantly negatively correlated with SBD_20_, SBD_40_ and SR (Table 7). It is clear that DT improved the soil structure and water and fertilizer retention properties of soil by promoting the rapid accumulation of SOC, increasing SCP and reducing NCP in the early stage of reclamation, thus increasing yield of crop. In the later stage of reclamation, DST can maintain a reasonable soil structure in the entire soil layer by reducing SBD and maintaining higher STP and SCP on the basis of higher SOC content, and balance the reasonable distribution of soil nutrients between the upper and lower soil layers by reducing the SR of SOC, thus promoting the high yield of crop.

## 4. Discussion

### 4.1. The Impact of Various Tillage Methods on SBD and Soil Porosity of Reclaimed Soil

Farmland management measures are the key factors affecting the evolution of soil quality, and unreasonable management will damage soil structure and lead to soil quality degradation [11]. SBD and soil porosity are important indexes reflecting soil structure. The influence of tillage method on soil structure is firstly reflected in the change in SBD. Different tillage methods have different effects on SBD. Compared with traditional tillage, NT can significantly increase SBD, especially SBD of the surface [15]. However, some studies believe that different tillage methods have a significant impact on SBD only in the 0–20 cm soil layer, and less impact on the soil layer below 20 cm [16]. This study also showed that different tillage practices had different effects on SBD of reclaimed farmland. The SBD of ST, DT and DST was significantly lower than that of NT. Tillage methods also directly affects soil porosity. NT not only increases SBD, but also reduces STP and SCP compared with traditional tillage, especially in some fields with low SBD [17]. In this study, the STP and SCP of each soil layer in ST, DT and DST were significantly higher than those in NT. This is mainly because ST and DT increase soil porosity through soil tillage. This is particularly due to these tillage methods using straw returning to the field which can keep the soil loose for a long time and further increase the porosity by mixing straw and soil [18]. In addition, in this study, the effects of different tillage methods on SBD and the soil porosity of each soil layer were significantly different with the increase in reclamation years. In the early stage of reclamation, ST had the lowest SBD and the highest STP and NCP in 0–20 cm soil layer, while DT had the highest SCP and the lowest NCP. In the 20–40 cm soil layer, DT had the lowest SBD and the highest STP and SCP, followed by DST. This is mainly because ST mixed the 0–20 cm soil layer with straw through tilling, thus reducing the SBD of the 0–20 cm soil layer and increasing its porosity. DT is the mixing of soil and straw in the deep soil layer (0–30 cm soil layer), which reduces the SBD and increases the soil porosity of the deep soil layer.

At the later stage of reclamation, DST has the best soil structure characteristics in all soil layers, namely the lowest SBD, the highest STP and SCP. Due to the long-term serious disturbance on soil structure, DT accelerated the mineralization decomposition of SOC in all soil layers, destroyed the soil aggregate structure, and finally affected the SBD and soil porosity. This is attributed to aggregate disruption under excessive disturbance, leading to increased SOC mineralization [19,20]. In contrast, DST provides moderate disturbance that alleviates compaction while preserving aggregate stability and SOC [21]. Therefore, the STP and SCP of DT were less than DST, but the NCP was greater than DST. It should be noted that soil structural indicators were not directly measured in this study; therefore, the discussion of the processes underlying soil physical changes is limited. Future studies will include additional indicators to better understand the effects of different tillage practices on the physical properties of reclaimed soils.

### 4.2. Effects of Different Tillage Measures on SOC and CSR

SOC is one of the main evaluation indexes of reclaimed land quality in mining areas, which is beneficial for improving soil aggregate structure and soil water and fertilizer retention capacity [2]. Previous studies have shown that SOC increases significantly in the early stages of reclamation after reclamation of deserts for farmland, but will reach a dynamic equilibrium or even decrease with the extension of farming years [22,23]. This study also showed that in the early stage of reclamation (2014–2017), the SOC content of each treatment increased rapidly with the increase in reclamation years, and gradually stabilized in the late stage of reclamation (2018–2021). This is because soil carbon sequestration is a dynamic cycle of SOC mineralization decomposition and carbon input. The reclaimed soil has very low SOC content compared with normal farmland soil, and its carbon sequestration potential is huge. Therefore, the carbon sequestration rate (CSR) is also faster than that of normal farmland soil [2,24]. With the continuous input of exogenous organic carbon, when the SOC in reclaimed soil increases to the state of “carbon saturation”, the CSR of soil will decrease [25]. This study also shows that with the continuous increase in SOC content, the CSR slowed down in the later period of reclamation. In addition, different tillage methods also have different effects on SOC because of the great difference in soil disturbance degree. In the early stage of reclamation, the CSR of ST, DT and DST were higher than that of NT. This is because, compared with NT, deep tillage and shallow tillage can bury straw into the soil, which can not only rapidly increase soil carbon storage, but also improve soil physical and chemical properties and promote crop growth, further leading to the increase in root carbon input in the soil. However, at the later stage of reclamation, the CSR of DT and DST at 0–20 cm was significantly lower than that of NT and ST. This is because long-term deep tillage destroys soil structure, exposes more soil aggregates, accelerates the mineralization and decomposition of upper SOC, and then reduces the CSR of SOC.

The impact of tillage methods on SOC content varied across different soil layers. NT and ST cover organic materials on the surface of the soil, which promoted the accumulation of SOC in the surface soil, but have little impact on the lower soil [26]. However, DT and DST could bring straw into deep soil, which increased the exogenous carbon input of deep soil, resulting in the increase in SOC content in the whole soil layer. Therefore, the SOC content in each soil layer of DT and DST is greater than that of ST and NT in the initial phase of reclamation. During the later stage of reclamation, SOC content of DT was significantly lower than that of ST and DST in 0–20 soil layer, while DT and DST had higher SOC content in the 20–40 cm soil layer. This is because SOC gradually approached saturation state with the increase in reclamation years. Although DT can significantly improve the content of SOC in the deep soil, it destroys the upper soil structure, exposes more soil aggregates, and accelerates the mineralization decomposition of SOC in surface soil [27]. Shallow tillage (ST) can reduce soil disturbance and effectively control the loss of surface SOC [28]. Therefore, ST has higher SOC content in the 20 cm soil layer [29]. Long-term NT mainly increased SOC content in 0–10 cm soil layer [30,31,32], but had little effect on SOC below 10 cm soil layer [33] and even reduced its content [34]. Therefore, the average SOC content of the 0–20 cm soil layer under NT was lower than that of other treatments. DST can regulate the distribution of soil organic carbon (SOC) and nutrients between upper and lower soil layers by promoting their redistribution across the soil profile, thereby avoiding excessive accumulation in the surface layer and contributing to improved soil structure [35]. Prior research has demonstrated that, compared with single tillage, rotation tillage can effectively coordinate the distribution of SOC in the 0–60 cm soil layer, thus improving the SOC content throughout the entire soil profile [36]. This study also showed that long-term DST significantly increased the SOC content of surface soil compared with DT, and increased the SOC content of deep soil compared with ST. It is clear that DT plays a significant role in the rapid increase in SOC content of each layer during the initial phase of reclamation, while NT and ST only increase the surface SOC content, but have little effect on the SOC of deep soil layer. Long-term DST can improve the SOC content of each soil layer, help to achieve long-term carbon sequestration goal, and meet the needs of agricultural sustainable production in reclaimed land.

### 4.3. Effects of Various Tillage Methods on SR of SOC

Although SOC can serve as an evaluation index of soil quality, it is inadequate for evaluating the influence of different tillage measures on soil quality. SR of SOC can represent the changes in soil quality, which can serve as an evaluation index to judge the direction of soil succession [4], and soil quality can be evaluated more comprehensively by studying SOC stratification [37]. Therefore, analyzing SR of SOC is helpful to understand the influence of different tillage measures on the quality of reclaimed land. This study showed that different tillage practices had different effects on SR of SOC. During the early reclamation period, the SR of SOC in each treatment showed an increasing trend. This is because, in the early stage of reclamation, the organic carbon released by straw returning to the field is rapidly accumulated in the surface soil, resulting in a gradual increase in SR of SOC. With the increase in reclamation years, the SR of DT and DST decreased significantly in the late reclamation period, while the SR of NT and ST remained high. This is because the SOC in the surface soil approaches or reaches the state of “carbon saturation” with the increase in reclamation years, but DT and DST increase the SOC content of deep soil layer, so the SR of DT and DST are significantly lower than NT and ST in the later reclamation period. Previous studies have also shown that long-term DT can reduce the SOC content in the surface layer and increase the SOC in the deep layer, thus reducing SR of SOC [38]. However, NT and ST can increase the SR of SOC by leaving straw on the soil surface and enriching nutrients on the surface [39,40].

### 4.4. The Impact of Various Tillage Methods on the Productivity of Reclaimed Soil

Different tillage practices can affect soil structure and SOC content, which can affect soil water and fertilizer retention performance and crop production. Reasonable tillage measures can delay the decomposition of organic matter in soil, increase SOC and nutrient content, and thus improve crop yield, and the improvement of crop yield often indicates soil improvement [41,42]. In this study, as the number of reclamation years increases, the crop yield of each treatment showed an upward trend, indicating that soil quality was gradually improved. However, different tillage measures had varying influence on the yield through affecting soil structure and SOC content at different reclamation stages. In the early stage of reclamation, ST, DT and DST had higher yields than NT. DT had the highest yield, followed by DST. This is because the reclamation land had poor soil structure, and the performance of water and fertilizer retention in the early stage and deep tillage combined with straw returning is more beneficial to SOC accumulation and soil structure improvement, so as to improve soil water and fertilizer holding capacity and crop yield. Long-term DT will damage soil structure, promote soil carbon mineralization and decomposition, and then affect SOC storage and crop production. However, rotating tillage can improve SOC and nutrient content by maintaining soil structure stability, thus achieving high yield [43]. In this study, the yield and increase rate of DT were lower than those of DST at the later stage of reclamation. This is because SOC reaches a gradual saturation state in the late stage of reclamation, and due to the large disturbance of DT on the soil, the negative impact of DT on soil structure gradually increases with the increase in reclamation years, which is not conducive to SOC accumulation and the continuous high yield of reclaimed cultivated land; this is also similar to the study of Soltanabadi et al. [44], but in their study DST was more conducive to the improvement of reclaimed soil quality and the sustainability of crop production since it reduced soil disturbance and increased soil SOC content. In addition, the correlation analysis also showed that DT in the early stage of reclamation improved the soil structure and water and fertilizer retention capacity of soil by promoting the rapid accumulation of SOC, increasing SCP and reducing NCP, thus increasing crop yield. In the later stage of reclamation, DST can maintain a reasonable soil structure of the entire soil layer by reducing SBD and maintaining higher STP and SCP on the basis of higher SOC content, and balance the reasonable distribution of soil nutrients between the upper and lower soil layers by reducing the SR of SOC, thus promoting the high yield of crops. Although long-term ST can effectively improve surface SOC content, organic carbon enrichment in the surface tends to present a greater ecological risk [45]. In the late growth period of wheat, surface roots gradually age, and plants mainly rely on deep roots to absorb nutrients and water. In the case of topsoil drought, surface accumulation of soil nutrients may limit the absorption and utilization of deep nutrients and water by plants, and ultimately affect the yield [46]. In addition, although macronutrients such as N, P, and K were not directly measured in this study, different tillage practices may alter their distribution within the soil profile by affecting soil structure. Deep tillage promotes the redistribution of nutrients into deeper layers, whereas shallow tillage and no tillage tend to concentrate nutrients in the surface soil. This may influence nutrient utilization by crops and contribute to differences in yield among treatments [47,48]. In this study, although ST had the highest SOC content in the surface soil at the later stage of reclamation, the low SOC content and large NCP in the deep soil affected the water and fertilizer retention performance of the deep soil, thus affecting the absorption and utilization of water and fertilizer in the deep soil by crops, meaning that the yield of ST was significantly lower compared to DT and DST. DT improves the SOC content in deep soil, but long-term DT reduces SOC in the whole soil layer, resulting in lower soil water and fertilizer retention ability compared to DST. Therefore, the yield of DT was significantly lower than that of DST in the late reclamation period.

It should be noted that statistical comparisons in this study were conducted among treatments within the same year and soil layer. While this approach effectively reflects temporal changes among treatments, it may not fully capture the interaction effects among factors or the relationships among soil structural properties, SOC dynamics, and crop yield. Future studies could apply methods such as two-way ANOVA to more systematically analyze these factors and their interactions, thereby providing a clearer understanding of how different tillage practices affect soil properties, SOC, and crop yield.

## 5. Conclusions

(1) Different tillage methods had various effects on the SBD and soil porosity of each soil layer with the increase in reclamation years. In the early stage of reclamation, DT rapidly improves soil structure by increasing SCP and reducing NCP in each soil layer. With the increase in reclamation years, DST helps to maintain lower SBD and higher STP and SCP, so that each soil layer has better soil structure characteristics in the late stage of reclamation.
(2) Various tillage methods had varying effects on the accumulation of SOC due to their influence on CSR. DT rapidly increases the SOC of the 0–40 cm soil layer in the early stage of reclamation, and DST facilitates maintaining higher SOC in the later stages. As compared to DT and DST, ST contributed more to SOC accumulation in surface soil, but less to SOC accumulation in deep soil.
(3) The impacts of different tillage techniques on the SR of SOC also exhibited considerable variation. NT had the lowest SR in the early stage of reclamation. Nevertheless, NT and ST maintained their high SR in the subsequent stage, whereas the SR of DT and DST experienced a notable decline due to the increase in SOC in deep soil.
(4) Additionally, the correlation analysis demonstrated that DT in the early stage of reclamation improved the soil properties by promoting the rapid accumulation of SOC, increasing SCP and reducing NCP, thus increasing crop yield. In the later stage, DST can maintain a better soil structure of the entire soil layer by reducing SBD and maintaining higher STP and SCP, and can balance the reasonable distribution of soil nutrients between the upper and lower soil layers by reducing the SR of SOC, thus promoting the high yield of crop.

## Figures and Tables

**Table 1:** Basic physical and chemical properties of soil.

Soil Depth/(cm)	Mass Fraction (g kg^−1^)				pH	Bulk Density /(g cm^−3^)
	Organic Carbon	Total N	Total P	Total K		
0–20	3.8	0.52	0.42	1.75	7.9	1.26
20–40	3.3	0.35	0.36	1.68	8.0	1.27
40–60	3.1	0.31	0.28	1.52	8.1	1.29

**Table 2:** Change in SOC (g kg^−1^) of different tillage methods.

Years	0–20 cm				20–40 cm			
	NT	ST	DT	DST	NT	ST	DT	DST
2014	3.83 ± 0.12 c	4.42 ± 0.14 b	5.02 ± 0.21 a	4.91 ± 0.13 a	3.35 ± 0.11 b	3.91 ± 0.09 a	4.32 ± 0.12 a	4.22 ± 0.08 a
2015	4.72 ± 0.13 c	5.46 ± 0.12 b	6.24 ± 0.22 a	5.93 ± 0.18 a	4.06 ± 0.08 b	4.52 ± 0.11 a	4.74 ± 0.12 a	4.67 ± 0.12 a
2016	5.84 ± 0.09 c	6.61 ± 0.11 b	7.87 ± 0.23 a	7.32 ± 0.23 a	4.43 ± 0.12 c	4.73 ± 0.16 b	5.22 ± 0.19 a	4.93 ± 0.17 ab
2017	6.87 ± 0.23 c	8.23 ± 0.23 b	8.83 ± 0.28 a	8.64 ± 0.27 ab	4.72 ± 0.10 c	5.13 ± 0.11 b	5.63 ± 0.15 a	5.42 ± 0.13 ab
2018	7.81 ± 0.28 c	9.40 ± 0.22 a	9.23 ± 0.21 a	8.83 ± 0.27 b	5.14 ± 0.22 c	5.72 ± 0.32 b	6.13 ± 0.21 ab	6.35 ± 0.19 a
2019	9.16 ± 0.23 c	10.41 ± 0.26 a	9.84 ± 0.32 b	9.97 ± 0.34 b	5.33 ± 0.11 c	6.31 ± 0.15 b	6.72 ± 0.19 a	6.92 ± 0.13 a
2020	9.43 ± 0.22 b	10.63 ± 0.29 a	9.72 ± 0.25 b	10.52 ± 0.32 a	5.64 ± 0.12 c	6.26 ± 0.09 b	6.83 ± 0.19 a	7.16 ± 0.23 a
2021	9.67 ± 0.33 c	10.84 ± 0.23 a	9.93 ± 0.25 c	10.40 ± 0.21 a	5.80 ± 0.17 c	6.63 ± 0.12 b	6.91 ± 0.24 b	7.34 ± 0.20 a

NT: no tillage; DT: deep tillage; ST: shallow tillage, DST: DT-ST alternating tillage. Different letters in the same soil layer of the same year show significant differences among treatments (*p* < 0.05).

**Table 3:** SOC storage (t C ha^−1^) under different tillage treatments from 2014 to 2021.

Years	0–20 cm				20–40 cm			
	NT	ST	DT	DST	NT	ST	DT	DST
2014	10.49 ± 0.37 c	11.93 ± 0.44 b	13.29 ± 0.59 a	13.02 ± 0.43 a	9.45 ± 0.33 b	10.95 ± 0.31 a	12.10 ± 0.37 a	11.72 ± 0.26 a
2015	13.85 ± 0.43 d	15.14 ± 0.49 c	17.85 ± 0.69 a	16.86 ± 0.58 b	12.02 ± 0.28 b	13.20 ± 0.40 a	13.46 ± 0.38 a	13.46 ± 0.39 a
2016	17.29 ± 0.33 c	18.52 ± 0.36 b	22.90 ± 0.75 a	21.00 ± 0.71 ab	13.29 ± 0.40 c	14.00 ± 0.41 bc	14.93 ± 0.59 ab	14.40 ± 0.54 b
2017	20.05 ± 0.74 c	22.68 ± 0.75 b	24.93 ± 0.87 a	24.01 ± 0.80 ab	14.73 ± 0.37 c	15.60 ± 0.43 bc	16.33 ± 0.51 a	16.04 ± 0.47 ab
2018	22.46 ± 0.87 c	25.58 ± 0.99 a	24.89 ± 0.76 ab	23.63 ± 0.79 b	15.83 ± 0.68 c	17.27 ± 0.98 b	17.90 ± 0.71 ab	18.16 ± 0.59 a
2019	26.38 ± 0.75 b	28.09 ± 0.86 a	25.78 ± 0.95 c	25.92 ± 0.94 bc	16.31 ± 0.39 c	18.93 ± 0.56 b	19.49 ± 0.62 ab	19.65 ± 0.45 a
2020	26.39 ± 0.71 b	28.45 ± 0.83 a	25.63 ± 0.81 c	27.08 ± 0.91 ab	17.37 ± 0.46 c	18.91 ± 0.40 b	19.94 ± 0.66 ab	20.33 ± 0.73 a
2021	26.66 ± 0.98 b	28.92 ± 0.76 a	25.89 ± 0.84 c	26.58 ± 0.61 bc	17.86 ± 0.60 c	19.76 ± 0.49 b	20.04 ± 0.76 ab	20.70 ± 0.63 a

NT: no tillage; DT: deep tillage; ST: shallow tillage, DST: DT-ST alternating tillage.

**Table 4:** Change in CSR (t C ha^−1^ a^−1^) in different tillage treatments.

Reclamation Stages	0–20				20–40			
	NT	ST	DT	DST	NT	ST	DT	DST
Early stage	2.37 ± 0.15 d	3.02 ± 0.21 c	3.59 ± 0.26 a	3.36 ± 0.22 b	1.40 ± 0.12 d	1.61 ± 0.15 c	1.80 ± 0.18 a	1.72 ± 0.13 b
Late stage	1.65 ± 0.13 a	1.56 ± 0.14 b	0.24 ± 0.05 d	0.64 ± 0.09 c	0.78 ± 0.03 d	1.04 ± 0.09 b	0.93 ± 0.07 c	1.17 ± 0.14 a

NT: no tillage; DT: deep tillage; ST: shallow tillage, DST: DT-ST alternating tillage. Different letters at the same growth stage show significant differences among treatments (*p* < 0.05). Early stage (2014–2017); late stage (2018–2021).

**Table 5:** Change in soil carbon SR in different tillage treatments.

Treatments	2014	2015	2016	2017	2018	2019	2020	2021
NT	1.15 ± 0.08 a	1.18 ± 0.04 b	1.32 ± 0.12 b	1.45 ± 0.07 b	1.53 ± 0.10 ab	1.72 ± 0.06 a	1.69 ± 0.08 a	1.64 ± 0.07 a
ST	1.12 ± 0.05 a	1.2 ± 0.06 b	1.42 ± 0.10 ab	1.61 ± 0.11 a	1.65 ± 0.11 a	1.65 ± 0.09 a	1.71 ± 0.07 a	1.64 ± 0.12 a
DT	1.17 ± 0.13 a	1.32 ± 0.04 a	1.5 ± 0.04 a	1.57 ± 0.06 a	1.51 ± 0.03 b	1.40 ± 0.06 b	1.43 ± 0.11 b	1.42 ± 0.08 b
DST	1.16 ± 0.10 a	1.29 ± 0.09 a	1.49 ± 0.12 a	1.59 ± 0.05 a	1.39 ± 0.13 c	1.43 ± 0.09 b	1.49 ± 0.05 b	1.47 ± 0.06 b

NT: no tillage; DT: deep tillage; ST: shallow tillage, DST: DT-ST alternating tillage. Different letters in the same year show significant differences among treatments (*p* < 0.05).

**Table 6:** Changes in grain yield (kg hm^2^) under different tillage measures.

Treatments	Reclamation Years							
	2014	2015	2016	2017	2018	2019	2020	2021
NT	3450.9 ± 124.3 c	4110.3 ± 174.2 d	4515.6 ± 182.1 d	4815.2 ± 112.3 d	5220.9 ± 204.4 c	5370.6 ± 219.1 d	5565.6 ± 189.2 d	5700.6 ± 193.2 d
ST	3600.2 ± 108.2 b	4395.9 ± 201.1 c	4830.4 ± 114.8 c	5130.7 ± 201.1 c	5700.6 ± 189.3 b	5775.7 ± 192.3 c	5790.4 ± 118.2 c	5970.8 ± 178.6 c
DT	3750.8 ± 122.3 a	4800.7 ± 98.9 a	5280.2 ± 142.3 a	5565.6 ± 112.3 a	6345.4 ± 183.4 a	6285.1 ± 123.3 b	6240.6 ± 178.3 b	6360.9 ± 125.6 b
DST	3780.7 ± 142.5 a	4650.8 ± 132.1 b	5085.5 ± 210.1 b	5385.0 ± 102.1 b	6270.5 ± 156.3 ab	6525.7 ± 119.6 a	6435.4 ± 196.3 a	6600.2 ± 154.7 a

Different letters in the same year imply significant differences between different treatments at *p* < 0.05. NT: no tillage; DT: deep tillage; ST: shallow tillage, DST: DT-ST alternating tillage.

**Table 7:** Correlation between yield and soil indexes.

Yield	SBD_20_	SBD_40_	STP_20_	STP_40_	SCP_20_	SCP_40_	NCP_20_	NCP_40_	SOC_20_	SOC_40_	SOC_T_	SR
2014–2017	0.360	0.303	−0.469 b	−0.388 b	0.943 a	−0.044	−0.901 a	−0.924 a	0.954 a	0.960 a	0.966 a	0.872 a
2018–2021	−0.911 a	−0.916 a	0.936 a	0.924 a	0.925 a	0.88 a	0.251	−0.272	0.467 b	0.840 a	0.714 a	−0.613 a

SBD_20_, SBD_40_ is soil bulk density in 0–20 cm, 20–40 cm soil layer, respectively; STP_20_, STP_40_ is soil total porosity in 0–20 cm, 20–40 cm soil layer, respectively; SCP_20_, SCP_40_ is capillary porosity in 0–20 cm, 20–40 cm soil layer, respectively; NCP_20_, NCP_40_ is non-capillary porosity in 0–20 cm, 20–40 cm soil layer, respectively; SOC_20_, SOC_40_ is SOC in 0–20 cm, 20–40 cm soil layer, respectively; SOC_T_ is SOC of the entire soil layer; SR is stratification ratio of organic carbon. a and b indicated significant correlation at *p* < 0.01 and *p* < 0.05 levels, respectively.

## Data Availability

Data will be made available on request.
